# Global Analysis of Extracytoplasmic Stress Signaling in *Escherichia coli*

**DOI:** 10.1371/journal.pgen.1000651

**Published:** 2009-09-18

**Authors:** Stéphanie Bury-Moné, Yanoura Nomane, Nancie Reymond, Romain Barbet, Eric Jacquet, Sandrine Imbeaud, Annick Jacq, Philippe Bouloc

**Affiliations:** 1 Laboratoire de Signalisation et Réseaux de Régulations Bactériens, Université Paris-Sud 11, CNRS, UMR8621, Institut de Génétique et Microbiologie Bâtiment 400, Orsay Cedex, France; 2 Centre de Génétique Moléculaire, UPR2167 CNRS, Gif/Orsay DNA Microarray Platform (GODMAP), Université Paris-Sud 11, Gif sur Yvette, France; 3 Institut de Chimie des Substances Naturelles, UPR2301 CNRS, IMAGIF qPCR-Platform, Gif sur Yvette, France; Université Paris Descartes, INSERM U571, France

## Abstract

The Bae, Cpx, Psp, Rcs, and σ^E^ pathways constitute the *Escherichia coli* signaling systems that detect and respond to alterations of the bacterial envelope. Contributions of these systems to stress response have previously been examined individually; however, the possible interconnections between these pathways are unknown. Here we investigate the dynamics between the five stress response pathways by determining the specificities of each system with respect to signal-inducing conditions, and monitoring global transcriptional changes in response to transient overexpression of each of the effectors. Our studies show that different extracytoplasmic stress conditions elicit a combined response of these pathways. Involvement of the five pathways in the various tested stress conditions is explained by our unexpected finding that transcriptional responses induced by the individual systems show little overlap. The extracytoplasmic stress signaling pathways in *E. coli* thus regulate mainly complementary functions whose discrete contributions are integrated to mount the full adaptive response.

## Author Summary

Bacteria possess various signaling systems that sense and respond to environmental conditions. The bacterial envelope is at the front line for most external stress conditions; its components sense perturbations and transmit signals to induce transcriptional reprogramming, leading to an adaptive response. In *Escherichia coli*, at least five response pathways, called Bae, Cpx, Psp, Rcs, and σ^E^, are induced in response to envelope stress. To date, these pathways have been studied mainly individually, and the interconnections and/or overlaps between them have not been extensively characterized. The present study establishes two important characteristics of stress response in *E. coli*: first, that a given stress solicits the combined responses of several pathways; second, that each individual pathway controls a discrete set of genes involved in the response, and shows little overlap with other pathways. Based on previous knowledge and the present data, we propose that an environmental stress probably impacts on the cell envelope by inducing numerous alterations, each of which may be perceived by different pathways of the stress response and contributes to adapting the cell to different aspects of the stress damage. The extracytoplasmic stress signaling pathways in *E. coli* thus regulate mainly complementary functions whose discrete contributions are integrated to mount the full adaptive response.

## Introduction

Bacteria possess various stress signaling systems that sense and respond to specific stimuli and allow the cell to cope with changing environmental conditions. One or several stress stimuli may activate multiple stress response pathways to constitute an integrated and complex response. Adaptation to envelope stress illustrates the complexity of these regulatory networks.

The bacterial envelope is involved in necessary processes including nutrient transport, respiration, secretion, adhesion, virulence and maintenance of bacterial integrity. In Gram negative bacteria such as *Escherichia coli*, the envelope comprises an inner membrane, a periplasmic space that contains the cell wall, an outer membrane and bacterial appendages such as pili and flagella. Being in contact with the external medium, the envelope is the initial target of physical (e.g., hyperthermia, osmolarity), chemical (e.g., ethanol, pH, detergent) or biological (e.g., adhesion, infection) stresses that may alter envelope components, thus inducing an extracytoplasmic stress response. The *E. coli* σ^E^, Psp, Cpx and Bae signaling pathways are the main elements of this response described to date (reviewed in [1]). The σ^E^ and Psp (phage shock protein) pathways are both regulated *via* sequestration and release of a transcriptional factor in response to specific signals: Accumulation of specific misfolded outer membrane proteins (OMP) within the periplasm induces sequential regulated intramembrane proteolysis (RIP) events leading to degradation of the inner membrane protein RseA, the σ^E^ sequestrator [2]–[4], and resulting in σ^E^ release in the cytoplasm. Free σ^E^ associates with RNA polymerase to allow σ^E^ -regulated gene transcription. PspF is a σ^54^ enhancer binding protein: In the absence of signals, PspF-enhanced transcription is inhibited by PspA binding to PspF [5]. According to the current model, one or both inner membrane proteins PspB and PspC sense the inducing signal (possibly a decrease of proton motive force) and then bind PspA, disrupting its interaction with PspF (reviewed in [6]). PspA, PspB and PspC thus act as regulators and effectors of the Psp response [7],[8], although another cascade might also exist [9]. The two other signal transduction pathways that respond to extracytoplasmic stress, Cpx for conjugative plasmid expression (for a review, see [10]) and Bae for bacterial adaptative response [11], are classical two component regulatory systems. Upon stimulation, the sensor (CpxA or BaeS) autophosphorylates a conserved histidine residue of its transmitter domain. The phosphoryl group is then transferred to a conserved aspartate of the receiver domain of the response regulator (CpxR or BaeR), resulting in its activation. In the absence of signals, sensor proteins are thought to function as phosphatases to deactivate their phosphorylated effector proteins. Additional proteins can participate in signal transduction prior to the sensor step: For example, the outer membrane lipoprotein NlpE stimulates CpxA following bacterial adhesion [12],[13], whereas the periplasmic protein CpxP inhibits CpxA autokinase activity in the absence of signal [14]. In the presence of an extracytoplasmic stress such as accumulation of P pili subunits, CpxP is titrated away from the CpxA periplasmic domain and degraded, together with bound misfolded proteins, by the periplasmic protease DegP [15]. P pili accumulation also induces the Bae pathway [11].

The Rcs system is a complex phosphorelay signaling pathway that also participates in the extracytoplasmic stress response. Initially described as a regulator of colanic acid capsule synthesis [16], mutational analyses later showed that the Rcs regulon also affects envelope composition [17],[18]. Recently, Rcs phosphorelay was shown to be activated by stresses affecting the peptidoglycan layer, and to contribute to intrinsic antibiotic resistance [19]. Rcs phosphorelay was also proposed to sense the extent of phosphorylation of the undecaprenyl carrier lipid, which is also involved in colanic acid synthesis [20],[21]. The Rcs pathway presents several differences as compared to classical two-component systems: RcsC is a hybrid sensor kinase having both a classical histidine kinase transmitter domain and an additional receiver domain with a conserved aspartate. Phosphate transfer from RcsC to RcsB is mediated by RcsD (formerly, YojN), a histidine-containing phosphotransmitter (Hpt). Finally, RcsB, the transcriptional regulator, utilizes an auxiliary cytoplasmic protein, RcsA, to regulate expression of some genes (for reviews, see [21]–[23]). Targets of the Rcs regulon can thus be classified as RcsA-dependent or RcsA-independent. RcsA is degraded by the Lon protease. Its instability has significant regulatory consequences, since the amount of RcsA is generally low. Formation of the RcsA-RcsB heterodimer protects RcsA from proteolysis and leads to transcriptional activation of RcsA-dependent genes and consequent capsule production.

The five pathways described above regulate chaperones, peptidyl-prolyl cis-trans isomerases, periplasmic disulfide isomerases, proteases that are involved in the folding or degradation of misfolded proteins, and also biosynthetic pathways for envelope components (reviewed in [24]). Together, these five envelope stress response systems are involved in the biogenesis, maintenance, and repair of the bacterial envelope, and thus; contribute to cell surface integrity. In addition, they modulate key bacterial life functions, such as motility (e.g., [25]–[27]), colony and biofilm formation (e.g., several *cpx*, *psp*, and σ^E^ regulated genes are induced during biofilm formation [28]; also see [12], [28]–[32]), conjugation [33],[34], stationary phase adaptation (e.g., [25], [35]–[38]), or virulence (e.g., [6], [36], [39]–[44], for reviews see, [1],[6],[45]).

The five envelope stress response systems have mainly been investigated individually. To gain further insights into the specificity of each pathway and their possible interconnections, here we compare the conditions leading to induction of each of the five pathways, and investigate the global transcriptional responses in parallel. This constitutes the first fully integrated transcriptomic study of extracytoplasmic stress response in *E. coli*.

## Results/Discussion

### Exogenous and genetic signals induce several extracytoplasmic stress pathways

Although molecular mechanisms leading to signal transduction and extracytoplasmic stress responses are well documented for several systems, the environmental conditions that act as natural inducers remain obscure. For each stress response system, several genetic defects or different treatments were shown to induce adaptive responses (i.e., Bae [11],[46]; Cpx [47]; Psp [6], [48]–[50]; Rcs [21],[22]; σ^E^ [4],[47],[50]). Some conditions are known to concomitantly activate several pathways. For instance, indole induces the Bae and Cpx pathways [11], ethanol, verapamil (calcium channel inhibitor), or dibucaine (an amide local anesthetic that alters membrane fluidity) induce the σ^E^ and Psp pathways [50], and antibiotics targeting penicillin binding proteins induce Rcs, Bae, Cpx and σ^E^ [19]. However the effects of activating signals have never been simultaneously compared for all five systems.

To determine whether a given stress condition could be specific to a single pathway, we investigated the impact of several stress conditions on activation of the five known extracytoplasmic stress response pathways. To do this, we first used strains with transcriptional gene fusions that place the *lacZ* reporter gene (encoding β-galactosidase) under the control of a promoter representative of each system: Bae, Cpx, Psp, Rcs and σ^E^ pathways were monitored using *spy*::*lacZ*, *cpxP*::*lacZ*, *pspA*::*lacZ*, *rprA*::*lacZ* and P3*rpoH*::*lacZ*, respectively (Table S1).

Strains were grown under various stress conditions using both external and genetic stimuli, and β-galactosidase activity was determined (see Materials and Methods). In the absence of their corresponding transcriptional regulators, *cpxP*::*lacZ* and *pspA*::*lacZ* fusions had no detectable activity and *rprA*::*lacZ* had strongly reduced activity when compared to the wild type background (data not shown). In the case of the σ^E^ reporter, P3*rpoH*::*lacZ*, σ^E^ is essential [51], and a basal level of β-galactosidase activity was observed in the unstressed condition.

Since the *spy*::*lacZ* fusion is dependent upon both BaeR and CpxR, we also analyzed the effects of these signals in a *baeR* background [52]. To our knowledge, no genes subjected only to *baeR* regulation have been described. We chose an *rprA*::*lacZ* reporter to monitor the Rcs pathway. *rprA* encodes a small regulatory RNA that stimulates *rpoS* (encoding σ^S^) translation [53]: This gene was used rather than a *cps*::*lacZ* fusion, since the latter fusion is also RcsA-dependent, and thus reports as much on the level of RcsA (which is potentially limiting) as on activation of RcsB [53].

None of the external tested stimuli were found to be strictly specific for a single signaling system (Table 1). Indeed, 5% ethanol and 4 mM indole induced all the transcriptional fusions tested. Dibucaine activated the Cpx, Rcs, Bae, and Psp systems whereas 0.6 M NaCl activated only the Rcs and Psp pathways. However, we observed differences in the response levels of reporter fusions to different signals: Cpx, Bae and σ^E^ pathways were induced preferentially by indole and ethanol, Rcs by NaCl in addition to ethanol and indole, and Psp by ethanol and dibucaine, in keeping with previous results [50]. Interestingly, the Rcs pathway was induced in all membrane-altering stress conditions tested, in accordance with a bona fide role of this pathway in extracytoplasmic stress response.

**Table 1:** Induction of extracytoplasmic stress response pathways in response to various stimuli^a^.

Pathways (Reporter fusions)	Genotype	Conditions							
		Indole 2 mM	Indole 4 mM	Ethanol 3%	Ethanol 5%	Dibucaine 0.5 mM	EDTA 5 mM	NaCl 0.6 M	pYedR
**Cpx** (*cpxP*::*lacZ*)	WT	+	+	++	+++	+	+	−b	+
**Rcs** (*rprA*::*lacZ*)	WT	+	++	++	+++	+	+	+++	+++
**Bae** (*spy*::*lacZ*)	WT *baeR*	+	+++	+++	+++	+	++	+	+
		−	+	+	+	−	++	+	+
**Psp** (*pspA*::*lacZ*)	WT	−	+	++	+++	+++	ND^c^	+	+++
σ^E^ (*rpoH*P3::*lacZ*)	WT	−	+	−	+	−	−	−b	−

a The induction level of the stress response pathways was determined using transcriptional gene fusion reporters expressing β-galactosidase. Cpx, Rcs, Bae, Psp, and σ^E^ pathways were tested using derivatives of strains TR50, GEB658, TR530, MC3, and CAG16037, respectively (Table S1). Results are presented as the ratio of β-galactosidase activity under stress conditions to standard conditions (growth in LB) from at least three independent experiments with the following codification: “+++”, ratio>5; “++”, ratio [3.5 ; 5[; “+”, ratio [1.5 ; 3.5[; “−”, ratio<1.5.

b β-galactosidase fusion less expressed than in standard conditions (LB).

c ND: not determined because the strain grew very poorly in this condition.

During a genetic screen using an *E. coli* genomic DNA library [54], we observed that overexpression of *yedR* (encoding a putative integral inner membrane protein conserved in enteric bacteria) led to a strong mucoid phenotype. This could indicate that YedR is a component of the Rcs pathway, or alternatively, that it acts as an internal inducer of the Rcs response. Further analysis showed that induction of capsule production by *yedR* depended on RcsB and RcsC proteins (data not shown). However, the *rprA*::*lacZ* fusion was responsive to different stresses (0.5 mM dibucaine, 3% ethanol or 0.6 M NaCl) in a Δ*yedR* background (data not shown), leading us to conclude that YedR is not part of the Rcs signal transduction pathway. Furthermore, multicopy *yedR* also strongly stimulated the Psp pathway, suggesting that YedR accumulation generates an envelope stress that is sensed preferentially by these two signaling systems (Table 1).

All the above results show that stress activates multiple pathways. Nevertheless, some signal are considered as specific activator of pathways: for example, e.g., exposure of the C-terminal part of certain OMPs to the PDZ domain of DegS activates σ^E^ [3],[55],[56], a drop of the proton motive force activates Psp [6], and accumulation of P-Pili subunits activates Cpx [13]; in the case of Rcs and Bae, specific signal sensing mechanisms remain to be identified. For Rcs, although it has been proposed that the signal could be a perturbation of the peptidoglycan [19], alteration in other envelope compartments can also be efficient inducers as previously reported [22] and illustrated by the impact of *yedR* overexpression (Table 1).

These observations may be reconciled, as the above tested conditions likely alter several aspects of bacterial envelope integrity, generating multiple signals that are in turn specifically sensed by different pathways. In addition, connections between stress response regulons could also account for indirect activation of some pathways.

In conclusion, one exogenous signal induces multiple defects by affecting different envelope components, which may be sensed by specific signal transducing mechanisms that activate all five pathways. It is expected that induction of all five extracytoplasmic stress responses is required to fully protect the cell against the variety of damages caused by a single stress.

### Extracytoplasmic stress signaling outputs: Parallel transcriptome analyses

To gain further insight into specifically regulated functions and interconnection between all five extracytoplasmic stress signaling systems, we carried out a global analysis of the general transcriptional responses following activation of each pathway. As discussed above, in view of the possible secondary signals generated by the use of inducers, we continued the study using an approach based on overexpression of each five pathway regulators. This methodology was previously used to characterize in detail the σ^E^ regulon [57]. We point out a possible limit to this approach, in cases where the regulator requires phosphorylation for its activity (i.e., for BaeR, RcsB, and CpxR); nevertheless, experimental evidence indicates that overproduction of such regulators can be effectively used in such studies, as a proportion of the molecules is phosphorylated in the absence of signal (e.g., [46],[58]).

*baeR*, *cpxR*, *rcsB*, *pspF* and *rpoE* were cloned under the control of the P_LtetO-1_ promoter in cloning vector pZE21, and the corresponding plasmids introduced into the wild-type strain MG1655Z1 (Table S1); a strain containing the plasmid with no insert was used as a control. Expression from the cloned genes was induced by addition of anhydrotetracycline (aTc). Since overexpression of CpxR [59], PspF (data not shown) and σ^E^ [35] was toxic, we determined the minimal aTc concentration that resulted in minimal cell toxicity. Accordingly, induction was carried out with 10 ng/mL aTc in exponential phase in LB medium at 37°C for 45 min, to limit indirect effects of regulator overexpression. Western blot experiments indicated that these conditions led to accumulation (approximately between three- to ten-fold) of CpxR, RcsB, and σ^E^ as compared to a strain having the control plasmid (data not shown; not determined for BaeR and PspF). Bacteria were harvested and total RNA was extracted for microarray experiments (Materials and Methods). In addition, 30 genes were selected to follow expression by qRT-PCR in six strains (overexpressing BaeR, CpxR, RcsB, PspF and σ^E^, or having the control plasmid). The qRT-PCR tested genes were chosen because: i) they were not previously known to be modulated during one of the studied conditions; ii) they were expected from published studies to be modulated by overexpression of one of the studied regulators, but the predictions were not confirmed by our micro-array experiments or, iii) the statistical significance of our micro-array data for these genes was inconclusive.

As detailed below, the increased transcription of *cpxR* resulted in induction of only a subset of genes previously shown to belong to the CpxR regulon. We therefore complemented these experiments with a transcriptome analysis of a *cpxR* mutant relative to the wild type MG1655 parental strain in late exponential phase. Since the Cpx pathway is activated in this condition [52],[59],[60], this comparison was expected to give access to at least some genes of the *cpxR* regulon.

#### The σ^E^ regulon

69 transcription units containing 114 genes were differentially expressed in response to σ^E^ overexpression. A vast majority (>90%) were induced, which is consistent with σ^E^ being a sigma factor (Table 2). Two previous studies reported an analysis of the σ^E^ regulon following transient overexpression [37],[57]. Our results are in good agreement with these studies; 35% of the operons that we found are in common with the two studies, and 64% are in common with one of the two studies. As previously described, and in keeping with the known function of the σ^E^ regulon, a large majority of the regulated genes are related to cell surface maintenance. Among the repressed genes, we found many that encode outer membrane proteins (*lamB*, *ompC*, *ompF*, *ompN*, *ompS1*, *ompW*, and *phoE*; Table 2) whose expression contributes to extracytoplasmic stress. Some of these genes are under the control of several sRNAs (many of which are also regulated by σ^E^) [61]–[66]. Among the σ^E^ up-regulated genes were those encoding sigma factors (*rpoD*, *rpoH*, *rpoN*), suggesting that regulatory cascades are triggered, as well as *ptsN*, a suppressor of *rpoE* essentiality when overexpressed [67]. As was previously reported [37],[57], the σ^E^ consensus binding site was not found upstream of several of the induced genes (e.g., the *imp-surA-pdxA* operon, and *djlA*, a gene divergent from *imp*, encoding a membrane associated co-chaperone of the DnaJ family [68],[69]). In the case of these genes, regulation by σ^E^ might be indirect, for instance by sRNAs, or by sigma factors that are increased by σ^E^ induction.

**Table 2:** Genes whose expression is significantly modulated by σ^E^.

Genes/putative operons^a^	bnum^b^	Product(s), operon description/function	Fold change(s)^c^
***degP^D K Rh^***	b0161	Periplasmic protease DegP	56.3 (63.9)
***ybfH^Rh^ ybfG***	b0691-0	Uncharacterized proteins	3.9; 25.2
***bamD^D Rh^***	b2595	Component of outer membrane assembly complex	14.6
***recC ptrA^Rh^recB^K Rh^ recD^Rh^***	b2822-19	PtrA: periplasmic protease III; RecBD: exodeoxyribonuclease V essential for recombination	4.5; 10.7; 4.5 (9.5); 3.1
***mdoG^D K Rh^ mdoH^Rh^***	b1048-9	Biosynthesis of membrane derived oligosaccharides	10.7; 4.2
***ycbK^K Rh^ ycbL***	b0926-7	YcbK: uncharacterized protein; YcbL: metal binding hydrolase.	9.7; 6.6
***eptB^K Rh^***	b3546	Phosphoethanolamine transferase, LPS biogenesis	8.5
***yhjJ^Rh^***	b3527	Periplasmic peptidase	8.0
***rseP^D Rh^ yaeT (bamA)^D Rh^ hlpA^D K Rh^ lpxD^D Rh^ fabZ^D K Rh^ lpxA^D K Rh^ lpxB^K Rh^*** * rnhB^Rh^ dnaE^Rh^*	b0176-84	RseP: inner membrane protease, regulator of the sigma E pathway; YaeT(BamA): Component of Outer Membrane Protein Assembly Complex; HlpA: periplasmic chaperone; Lpx A, B, D: biosynthesis of LipidA; FabZ: fatty acid elongation	7.4; 3,7; 1.7; 5.8; 2.2; 1.4; *1.7*; -; -
***yggN^D Rh^***	b2958	Uncharacterized protein, potentially involved in biofilm formation.	6.9
***htrG^D K Rh^ cca^K Rh^***	b3055-56	HtrG: Uncharacterized inner membrane protein; Cca: tRNA nucleotidyltransferase	2.1; 6.3
*kdsC * ***lptC(yrbK) lptA^Rh^ lptB^Rh^ rpoN^Rh^ hpf^Rh K^ ptsN^Rh K^ yhbJ^Rh K^ npr^K^***	b3198-06	LptAB-YrbK ABC transporter/LPS export; RpoN: σ^54^ transcription factor; Hpf: σ^54^ modulation protein; PtsN: phosphotransferase system enzyme IIA, regulates N metabolism; YhbJ: predicted P-loop containing ATPase; Npr: phosphocarrier protein HPr-like, nitrogen related.	-; 2.3; 6.0; 1.9; 2.4; 2.2; 2.3; 2.7; 1.4
***plsB^K Rh^***	b4041	Membrane-bound glycerol-3-phosphate acyltransferase/phospholipids biosynthesis	5.3
***yiiS^ReRh^ uspD^Re Rh K^***	b3922-23	YiiS: conserved protein ; UspD: stress protein involved in resistance to UV irradiation	1.8; 5.1
***ybhQ^K^***	b0791	Predicted inner membrane protein	5.0
***yfeK^Rh^ yfeS^Rh^***	b2419-20	Uncharacterized proteins	2.9; 4.8
***imp (lptD)^D K Rh^ surA^D K Rh^ pdxA^D Rh^ ksgA apaG apaH***	b0054-49	Imp (LptD): Component of outer membrane LPS assembly complex; SurA: periplasmid peptidyl-prolyl isomerase; PdxA: 4-hydroxy-L-threonine phosphate dehydrogenase; KsgA: S-adenosylmethionine-6-N′,N′-adenosyl (rRNA) dimethyltransferase; ApaG: uncharacterized protein; ApaH: diadenosine tetraphosphatase	2.5; 4.7; 2.3; 1.9; 1.6; 1.7
***diaA^Rh^ yraP^D Rh^***	b3149-50	DiaA: DnaA initiator-associating factor for replication initiation; YraP: lipoprotein, possible member of the DegP/Skp folding pathway	2.2; 4.5
*xerD^D K Rh^* ***dsbC^D K Rh^ recJ^D Rh^ prfB^K Rh^***	b2894-0	Periplasmic Disulfide prolyl isomerase; Single-stranded-DNA-specific exonuclease RecJ; Peptide chain release factor 2 (RF-2)	-; 4.0; 2.3; 4.3
***ymbA^Rh^***	b09452	Conserved lipoprotein YmbA	4.2
***ydbD***	b1407	Uncharacterized protein induced by methylglyoxal	3. 9
***matA***	b0294	Putative HTH-type transcriptional regulator	3.8
***smpA^Re K Rh^***	b2617	Component of Outer Membrane Protein Assembly Complex	3.6 (9.4)
*ybgC * ***tolQ tolR tolA^K^ tolB^K^*** * pal^K^* ***ybgF^K^***	b0736-42	Components of the Tol-Pal complex/envelope integrity; YbgF: unknown function, periplasmic	-; 3.5; 1.6; 2.7 (3.6); 2.5; -; 2.0
***yabI^K^***	b0065	Conserved inner membrane protein	3.4
***yfeY^Re K Rh^*** * yfeX^K Rh^*	b2432-1	Predicted outer membrane lipoprotein YfeY	3.3; -
*yhbC nusA infB rbfA * ***truB*** * rpsO^K^*	b3165-70	tRNA pseudouridine 55 synthase	-; -; -; -; 3.2; -
*dapA^D K^* ***bamC(nlpB)^D K Rh^***	b2478-77	BamC: component of Outer Membrane Protein Assembly Complex;	-; 3.1
***fkpA^D K Rh^***	b3347	Periplasmic peptidyl prolyl isomerase	3.1
***sspA*** * sspB*	b3229-8	Stringent starvation protein A	3.0; -
***yfgM^K Rh^ bamB^K Rh^ der^K Rh^***	b2513-1	Penicillin-binding protein 1C; BamB: component of Outer Membrane Protein Assembly Complex; Der: 50S ribosomal subunit stability factor	2.2; 3.0; 2.3
*yeaZ^Re^* ***yeaY^Re Rh^ fadD^Rh^ rnd***	b1807-4	YeaY: predicted lipoprotein; FadD: fatty acyl coA synthetase; RnaseD	-; 2.8; 2.2; 1.5
***mreB^Rh^ mreC^Rh^*** * mreD^Rh^*	b3251-49	Rod shape-determining proteins	1.6; 2.7; -
***degQ*** * degS*	b3234-5	Periplasmic protease DegQ	2.7; -
***djlA***	b0055	Inner membrane associated DnaJ-like protein	2.8 (6.2)
***rpoH^D Re K Rh^***	b3461	Sigma 32/heat-shock response	2.6
*yjeN * ***yjeO***	b4157-8	YjeO: Conserved inner membrane protein	-; 2.6
***yacG***	b0101	Conserved hypothetical protein with a zinc finger	2.6
***rseA^D K Rh^*** * rseB^D K Rh^ rseC^D Rh^*	b2572-0	RseA: Anti-sigma factor/regulation of σ^E^ activity	2.5; -; -
***ybhH***	b0769	Uncharacterized conserved protein	2.5
*rpsU * ***dnaG^D K^ rpoD^D K Rh^***	b3065-7	DNA primase; Sigma factor σ^D^	-; 1.7; 2.5
***yabP yabQ***	b0056-57	Uncharacterized proteins, genes downstream of *djlA*	2.5; 1.9
***purC***	b2476	Phosphoribosylaminoimidazole-succinocarboxamide synthase	2.4
***lpxP^Re K Rh^***	b2378	Palmitoleoyl acyltransferase, LPS biosynthesis	2.4
***yfgC^Rh^ yfgD^Rh^***	b2494-95	YfgC: TPR repeat-containing protein. Predicted periplasmic peptidase ; YfgD: predicted oxydoreductase	2.4; 1.5
***bacA^Re K Rh^***	b3057	Undecaprenyl pyrophosphate phosphatase/peptidoglycan byosynthesis	2.3
***yieE*** * yieF*	b3712-3	Uncharacterized protein	2.3; -
***yfiL***	b2602	Putative lipoprotein	2.3
***sbmA^Re K Rh^ yaiW^Re K Rh^***	b0377-8	Inner membrane transporter	2.2; 1.8
*proV proW * ***proX***	b2677-9	Glycine betaine-binding periplasmic protein	-; -; 2.1
*rhaA * ***rhaB*** * rhaD*	b3904-2	RhaB: rhamnulokinase, L-rhamnose degradation	-; 2.1; -
***yjdL***	b4130	Inner membrane transporter	2.0
***malQ^D K Rh^***	b3416	Amylomaltase, maltose catabolism	2.0
***yraQ***	b3151	Uncharacterized protein	2.0
***yjaB***	b4012	Conserved inner membrane protein	2.0
***yeiQ***	b2172	Predicted dehydrogenase, NAD-dependent	2.0
***groL***	b4143	GroL/Hsp60 chaperone	1.9
***yebA^K^ lpxM***	b1856-5	YebA: Uncharacterized metallopeptidase, inner membrane protein/cell wall degradation?; LpxM: Lipid A biosynthesis (KDO)2-(lauroyl)-lipid IVA acyltransferase	1.9; 1.4
***yaiY***	b0379	Inner membrane protein	−3.8 (16.3)^d^
***treB*** * treC*	b4240-39	PTS system trehalose-specific EIIBC components	−2.0; -
***ompW^J^***	b1256	Outer membrane protein	−2.3 (−*1.9*)
***tnaL*** *tnaA^K^* ***tnaB***	b3707-9	Tryptophan transport and utilization	−2.5; *−1.9* (−*2.7*); *−2.0*
***ompN^K^***	b1377	Outer Membrane porin N	−2.5
***yedS (ompS1)***	b1964	Outer membrane protein (Pseudogene?)	−2.5
***cspD^K^***	b0880	Cold shock protein homolog; DNA replication inhibitor CspD	−2.6 (−*1.53*)
***lamB***	b4036	Outer membrane porin, phage lambda receptor protein; maltose high-affinity receptor.	*−2.8 (−4.1)*
***ompC^Rh^***	b2215	Outer membrane porin OmpC	−2.8
***ompF^K Rh^***	b0929	Outer membrane porin OmpF	−2.8
***phoE^K^***	b0241	Outer membrane pore protein PhoE (Phosphate transport)	−3.3

a Genes are grouped by putative or known operons (ordered in the direction of transcription). Genes whose expression was found to be significantly modulated are in bold (see Materials and Methods). Genes whose expression was determined by Q-PCR are underlined, and the fold change thus determined is indicated in parentheses after the microarrays value. Genes previously reported to be σ^E^-regulated are indicated by uppercase letters that refer to the concerned study with the following codification: D, reported in [99]; K, reported in [37]; Re, reported in [100]; Rh, reported in [57]; and J, reported in [64].

b b numbers correspond to genes of the first column.

c The fold changes indicate the ratios of gene signal intensities of strains containing pZE21-rpoE to the reference signals (see Materials and Methods). Values correspond and follow the order of genes from the first column. The ratio value of genes not significantly modulated is indicated as “-”. Numbers in italics indicate a p-value>0.01 in case of microarrays or >0.05 in case of qRT-PCR.

d Microarray and qRT-PCR data concerning *yaiY* were divergent. We consider that *yaiY* results issued from microarray experiments are likely to be artifacts, possibly due to spotting of a non-*yaiY*–specific PCR product.

#### The RcsB regulon

Among genes whose expression is altered by RcsB overexpression, the vast majority (>90%) encodes proteins related to the envelope or its metabolism, or localized in the envelope (Table 3). All affected genes were induced, showing that RcsB is mainly a positive transcriptional regulator. Previous transcriptomic studies have explored Rcs regulated genes under different conditions: i) following DjlA overexpression in the absence of *rcsC* [30], ii) in exponential phase in the absence of *rcsB* or *rcsD* [70], and iii) in the absence of *rcsC* or *rcsF* at low temperature with or without zinc excess [71]. More than 70% of the genes that we identified as being differentially expressed after RcsB overexpression were also found in at least one of these studies; These include bacterial capsule synthesis genes (e.g., *wca-wza-cps*) or genes induced by an osmotic stress (*osmB* and *osmY*). The relatively low fold induction of the colonic acid synthesis operon in our conditions could be due to a limiting amount of RcsA. Additional genes of interest, previously not described, are involved in O antigen biosynthesis. qRT-PCR experiments also indicate that RcsB induces the expression of *spy* and *yaiY* genes encoding a periplasmic protein under the control of both BaeR and CpxR (see below) and a putative inner membrane protein, respectively (Table 3).

**Table 3:** Genes whose expression is significantly modulated by RcsB.

Genes/putative operons^a^	bnum^b^	Product(s), operon description/function	Fold change(s)^c^
***osmB^F H^***	b1283	Osmotically inducible lipoprotein	11.4
*ymgG^F^* ***ymgD^F^***	b1172-1	YmgD: putative periplasmic protein	-; 5.5
***osmY^F H^***	b4376	Hyperosmotically inducible periplasmic protein	4.2
***ybaY***	b0453	Predicted outer membrane lipoprotein	3.9
***ivy^F^***	b0220	Inhibitor of vertebrate lysozyme	3.4
***ycfJ^F^***	b1110	Uncharacterized putative inner membrane protein	3.2
***yfbR^F^***	b2291	dCMP phosphohydrolase	2.7
*yraI yraJ * ***yraK***	b3143-5	YraK: Putative fimbrial protein	-; -; 2.7
*glf * ***rfc***	b2036-5	Rfc: O-antigen polymerase/O-antigen biosynthesis	-; 2.4
***ugd^F^ cld^F^***	b2028-7	Ugd: UDP-glucose 6-dehydrogenase/colanic acid precursor biosynthesis; Cld: regulator of length of O-antigen	-; 2.4
***yfdC***	b2347	Predicted inner membrane protein	2.0
***yjbJ^F H^***	b4045	Predicted stress response protein, belongs to the σ^S^ regulon	2.0
***galU^F^***	b1236	GalU: Subunit of glucose-1-phosphate uridylyltransferase	2.0
***wza^F^*** * wzb^F^* ***wzc^F H^*** * wcaA^F H^ wcaB^H^*	b2062-58	Colanic acid biosynthesis and secretion	1.6; -; 1.9; -; -
***yaaX***	b0005	Putative periplasmic protein protein	1.9
*wcaC^F H^ wcaD^F H^* ***wcaE^F H^*** * wcaF^FH^* ***gmd^F^ fcl^F^*** * nudD^F H^ wcaI^FH^ cpsB^H^ cpsG^H^ wcaJ^H^ wzxC^F^*	b2057-46	WcaE: colanic acid biosynthesis glycosyl transferase; Gmd: GDP-mannose 4,6-dehydratase; Fcl: subunit of GDP-fucose synthase/Colanic acid biosynthesis and secretion	-; -; 1.5; -; 1.9; 1.7; -; -; -; -; -; -
***yggG^F H^***	b2936	Uncharacterized metalloprotease, liprotein	1.9
***yegS***	b2086	Lipid kinase	1.9
***spy***	b1743	Periplasmic protein related to spheroblast formation	*1.1* (3.9)
***yaiY^F^***	b0379	Inner membrane protein	−3.3 (17.9)^d^

a Genes are grouped by putative or known operons (ordered in the direction of transcription). Genes whose expression was found to be significantly modulated are in bold (see Materials and Methods). Genes whose expression was determined by Q-PCR are underlined, and the fold change thus determined is indicated in parentheses after the microarray value. Genes previously reported to be RcsB regulated are indicated by uppercase letters that refer to the concerned study with the following codification: F, reported in [30]; H, reported in [71].

b b numbers correspond to genes of the first column.

c The fold changes indicate the ratios of gene signal intensities of pZE21-rcsB containing strain to the reference signals (see Materials and Methods). Values correspond and follow the order of genes from the first column. The ratio value of genes not significantly modulated is indicated as “-” unless found significantly modulated by qRT-PCR. Numbers in italics indicate a p-value>0.01 in case of microarrays or >0.05 in case of qRT-PCR.

d Microarray and qRT-PCR data concerning *yaiY* were divergent. We considered that the *yaiY* results issued from microarray experiments are likely artifacts possibly due the spotting of a none *yaiY* specific PCR product.

#### The BaeR regulon

The Bae pathway is auto-regulated and specifically deals with toxic compounds by induction of the *mdt-bae* operon, which encodes a multidrug transporter of the RND family, and the *tolC* gene, which ensures efflux through the outer membrane ([72] and Table 4). Transcriptome analysis in conditions of BaeR overexpression provides the smallest gene list in this study. Indeed, up-regulation of only 8 genes, corresponding to 5 transcription units, was statistically significant, even considering a minimal ratio of induction of 1.4 confirming that like RcsB, BaeR acts mainly by stimulating transcription (Table 4). Only four BaeR binding sites, upstream of *spy*, the *mdt-bae* operon, *acrD*, and *ycaC*, have been reported in the *E. coli*. All genes were previously reported as having their expression activated by the Bae pathway [46],[70]; however, *spy*, *mdtA* and *mdtB* described as highly induced [46] were moderately induced in the microarray study (although *spy* was found to be upregulated more than 70 fold by qRT-PCR, variation in the expression of the other genes was not determined). This variation could reflect differences in parameters such as overexpression conditions, medium, growth phase, or method used (microarray technology tends to underestimate the variation of expression). It could also be due to (i) a role of CpxR in modulating BaeR activity [58],[73] (see also below), (ii) low affinity of BaeR for its target sequences, and/or (iii) differences in results depending on transient (our study) or constitutive overexpression [46]. We also observed induction of the *ynjABCD* operon that encodes a putative membrane transporter (Table 4). Induction of *ynjA* was confirmed by Northern blot (data not shown). Neither the *ynjABCD* operon nor the *tolC* gene displayed an upstream *baeR* binding consensus motif, suggesting that BaeR-mediated effects on these operons may be indirect.

**Table 4:** Genes whose expression is significantly modulated by BaeR.

Genes/operon^a^	bnum^b^	Product(s), operon description/function	Fold change(s)^c^
***mtdA^N^ mtdB^N^*** * mtdC^N^ mtdD^N^* ***baeS^N^*** * (baeR^N^)*	b2074-9	Subunits composition of MdtABC-TolC multidrug efflux transport system; BaeS: sensor of the Bae signal transduction pathway	5.3; 3.9; -; -; 1.5; -
***spy^O N^***	b1743	Periplasmic protein related to spheroblast formation	2.8 (74.8)
***tolC^N^*** * ygiA ygiB ygiC*	b3035-8	TolC outer membrane channel, subunit of MdtABC-TolC multidrug efflux transport system	1.9; -; -; -
***ynjA^N^ ynjB*** * ynjC ynjD*	b1753-6	Conserved proteins	1.6; 1.4; -; -
***yeeN^N^***	b1983	Conserved protein	1.4

a Genes are grouped by putative or known operons (ordered in the direction of transcription). Genes whose expression was found to be significantly modulated are in bold (see Materials and Methods). Genes whose expression were determined by Q-PCR are underlined, and the fold change thus determined is indicated in parentheses after the microarray value. Genes previously reported to be BaeR-regulated are indicated by uppercase letters that refer to the concerned study with the following codification: N, reported in [46]; O, reported in [70].

b b numbers correspond to genes of the first column.

c The fold changes indicate the ratios of gene signal intensities of pZE21-baeR containing strain to the reference signals (see Materials and Methods). Values correspond and follow the order of genes from the first column. The ratio value of genes not significantly modulated is indicated as “-”.

#### The Psp Regulon

PspF is a member of the enhancer-binding protein family of transcriptional regulators, which stimulates transcription by the alternative sigma factor σ^54^. PspA, encoded by the *pspABCDE* operon has a dual function. In normal conditions, it binds PspF, preventing transcription of the operon. But it is also thought to be the major effector of the Psp pathway, by promoting proton motive force maintenance in the inner membrane (for a review, see [6]). Only two previous studies addressed the nature and extent of the PspF regulon in *E. coli*. In one, overexpression of the pIV protein (a secretin from the filamentous phage f1) was shown to induce the *pspABCDE* operon, as well as an additional gene, *pspG* [48]. Subsequent transcriptome analyses were performed on *E. coli* strains disrupted for *pspA*, *pspD* and *pspG*. The *psp* genes, including *pspG*, were all found to be highly expressed in the *pspA* mutant [27]. In the present study, overproduction of PspF led to induction of the *pspABCDE* operon and *pspG* (Table 5) as previously reported [48]. These genes are directly involved in combating stress and/or regulating expression of the Psp pathway [7]. In addition, we identified 11 genes corresponding to 10 transcription units that were derepressed following PspF overproduction, and not previously reported (Table 5, [48]). Among them, *tolB* encodes a member of the Tol-Pal trans-envelope complex, which is required to maintain cell envelope integrity [74]. Interestingly, the interaction between TolA and the Pal lipoprotein is driven by the proton motive force [74]. Another significantly derepressed gene was *hyfR*, which controls expression of genes responsible for the proton-translocating formate hydrogenase system and formate transport. Derepression of the above functions is in keeping with the implication of PspF in maintaining bacterial proton motive force. *norW*, which encodes a flavorubredoxin reductase important for nitric oxide reduction and detoxification that is regulated by σ^54^ (RpoN) [75], was also strongly up-regulated (Table 5). Since PspF is a σ^54^ enhancer-binding protein [5], its overproduction may lead to a strong transcription of *norW*.

**Table 5:** Genes whose expression is significantly modulated by PspF.

Genes/operon^a^	bnum^b^	Product (s), operon description/function	Fold change(s)^c^
***pspA^L^ pspB^L^ pspC^L^*** * pspD^L^ pspE^L^*	b1303-8	Phage shock proteins	11.8; 3.6; 2.4; -; -
*tolA* ***tolB*** * pal ybgF*	b0739-42	Members of the Tol-Pal cell envelope complex/envelope integrity	1.8 (1.2); 8.2; -; -
*hyfABCDEFGHIJ * ***hyfR*** * focB*	b2491	HyfR transcriptional activator/controls the expression of genes responsible for the proton-translocating formate hydrogenase system and for formate transport under anaerobic condition	-; -; -; -; -; -; -; -; -;- ; 6.5; -
*norV * ***norW***	b2710-1	NorW: Flavorubredoxin reductase	-; 3.1 (125.8)
***yfbS***	b2292	Putative uncharacterized transport protein (inner membrane protein)	2.7
*ypdA ypdB * ***ypdC***	b2380-2	YpdC: Predicted AraC-type regulatory protein	-; -; 2.2
***hslV hslU***	b3932-1	ATPase component of the HslVU protease	1.4; 2.1
***yjiY***	b4354	Inner membrane protein	2.0
***pspG***	b4050	Phage shock protein G, inner membrane protein	2.0
*atoS * ***atoC***	b2219-20	Response regulator, acetoacetate metabolism regulation	-; 1.9
***nagB*** * nagA nagC nagD*	b0678-5	N-acetylglucosamine degradation	−2.0; -; -; -; -
***narK*** * narG^J^* ***narH*** * narJ narI * ***ychS***	b1223-8	NarK MFS nitrate/nitrite transporter, nitrate reductase A	−1.6; -; −1.4; -; -; −2.1
***lamB***	b4036	Outer membrane porin; Phage lambda receptor protein; maltose high-affinity receptor	−*2.1* (−13.6)
***ydeN***	b1498	Putative arylsulfatase	−2.1 (−*6.8*)
***glpF^J^ glpK^J^*** * glpX*	b3927-5	Glycerol facilitator, glycerol kinase/glycerol utilization	−2.2; −1.9; -
*napFD* ***^J^napA*** * napGHB * ***napC ccmA*** *BCDEFGH*	b2208-2194	NapABC: periplasmic nitrate reductase; CcmABC protoheme IX ABC transporter/anaerobic respiration of nitrate	-; -; −2.4 (−*2.7*); -; -; -;−1.4; −1.4 (−*1.7*); -; -; -; -; -; -; -
***raiA***	b2597	Stationary phase translation inhibitor and ribosome stability factor, induced in stationary phase and by cold shock	−2.5
***leuL^J^***	b0075	Leu operon leader peptide/regulation of leucine biosynthesis	−2.6
***cspD***	b0880	DNA replication inhibitor, induced by stress and glucose starvation, similarity to cold shock protein	−2.7 (*−2.6*)
***glpT glpQ***	b2240-39	Glycerol 3-phosphate transporter (MFS family); glycerophosphoryl diester phosphodiesterase, periplasmic.	−1.8; −2.7
***glpA glpB glpC***	b2241-3	Glycerol 3-phosphate deshydrogenase (anaerobic)	-; -; −2.9 (−*9.6* )
***tnaL tnaA tnaB***	b3707-9	Tryptophan utilization	−3.5; −3.5 (−113) ; −3.8

a Genes are grouped by putative or known operons (ordered in the direction of transcription). Genes whose expression was found to be significantly modulated are in bold (see Materials and Methods). Genes whose expression was determined by Q-PCR are underlined, and the fold change thus determined is indicated in parentheses after the microarrays value. Genes previously reported to be PspF-regulated are indicated by uppercase letters that refer to the concerned study with the following codification: J, reported in [27]; L, reported in [48].

b b numbers correspond to genes of the first column.

c The fold changes indicate the ratios of gene signal intensities of pZE21-pspF containing strain to the reference signals (see Materials and Methods). Values correspond and follow the order of genes from the first column. The ratio value of genes not significantly modulated is indicated as “-”. Numbers in italics indicate a p-value>0.01 in case of microarrays or >0.05 in case of qRT-PCR.

Several genes/operons were also found to be repressed in PspF overproduction conditions. In particular, *glpABC*, encoding glycerol-3-phosphate dehydrogenase, is down regulated. Consistent with this observation, these genes were up regulated in a strain depleted for *pspA* and/or *pspD* [27], whereas in our conditions, *pspA* was overexpressed 12-fold. Note that this level of induction is within the range observed by Lloyd et al [48] after overproduction of the filamentous phage f1 protein IV. Previous transcriptome studies indicated that Psp responds to decreased proton motive force due to membrane perturbations [27]. Lower *glpABC* in PspF overproduction conditions could reduce utilization of glycerol-3-phosphate as an electron donor for the respiratory chain, and help maintain the pool of glycerol-3-phosphate required for phospholipid synthesis. Glycerol-3-phosphate is a substrate of glycerol-3-phosphate acyltransferase encoded by *pls*, itself a gene under the positive control of σ^E^ (Table 2).

Also repressed were several genes encoding functions related to the anaerobic respiration of nitrate (i.e., several *nar* and *nap* genes) as well as genes required for cytochrome c biogenesis (*ccmA*). These results do not support the conclusion of Jovanovic et al [27] that the *psp* regulon responds to the dissipation of proton motive force by favoring anaerobic respiration of nitrate. In addition, contrary to their suggestion that a function of the Psp pathway was to down-regulate motility and chemotaxis, motility genes were conspicuously unaffected in our study (Table 5). These differences could be due to the fact that PspA induction was about 8-fold lower in our study than in the one previously reported [27].

#### The CpxR Regulon

Previous characterization of the CpxR regulon consisted mainly of using a CpxR sequence recognition weight matrix and identifying reliable target promoters within the *E. coli* genome [52]. This search identified genes that were directly regulated by CpxR, either positively or negatively. In addition, transcriptome analysis following a copper stress revealed twelve genes that were depressed by copper in a Cpx dependent manner [76]. Regulation of many of those genes was confirmed by a recent study using *lux* fusions in a MC4100 cpxA* background, where the Cpx pathway is constitutively activated [77]. This study also showed that the presence of a CpxR box upstream of the gene (or operon) was not a good predictor of the extent of regulation by CpxR.

We aimed to generate *in vivo* data supporting these predictions and observations, and investigated transcriptome modifications following CpxR overexpression in exponential phase. Results were surprising, as well-known targets of CpxR, such as *degP/htrA*, or *dsbA* [78] were not significantly regulated upon *cpxR* induction. *cpxP* and *spy* were derepressed at a level insufficient to be included in our gene selection (1.4 and 1.8 fold, respectively). Analysis of gene expression by qRT-PCR, which is more sensitive than microarrays, showed a two-fold induction of *dsbA*, a three-fold induction of *cpxP*, and strong induction of *spy* (40 times), but no induction of *degP* or *smpA* (Table 6). In the latter two cases, this could be explained by the dependence of gene transcription upon σ^E^, whose amount, in the absence of an inducing signal, might be limiting. To complement our data, we performed a comparative transcriptome analysis of MG1655 and its Δ*cpxR* derivative in late exponential phase, a condition in which the Cpx pathway is proposed to be activated [52],[59],[60]. Deletion of *cpxR* did not have a strong effect on most of the known *cpxR*-regulated genes (the maximum effect was 4 fold, see Table 7), as recently reported [77]. However, we found a four-fold reduction of expression of *yccA*, a modulator of the membrane protease HflB, a 2.5 reduction of *smpA* expression, and a 2.2 fold reduction of *ftnB*, encoding a ferritin-like protein.

**Table 6:** Genes whose expression is significantly modulated by CpxR.

Genes/putative operon^a^	bnum^b^	Product(s) description/function	Fold change(s)^c^
***yebE^Y^***	b1846	Conserved inner membrane protein	9.8
***yiaF***	b3554	Putative inner membrane lipoprotein	5.9 (7.71)
***yhaH yhaI yhaJ***	b3103-5	Predicted inner membrane protein; putative inner membrane protein; predicted DNA binding transcriptional regulator	1.9; 3.2 (40.3); 2.0
***yafK***	b0224	Conserved protein (periplasmic)	3.0
*gspC gspD * ***gspE*** * gspFHIJKLMO*	b3324-35	General secretory pathway component, cryptic	-; -; 2.7; - ; -; -; -; -; -; -; -
***ybgC tolQ*** * tolR * ***tolA tolB*** * pal * ***ybgF***	b0736-42	YbgC: predicted acyl –coA thioesterase; TolQRAB-YbgF: components of the Tol-Pal cell envelope complex	1.8; 2.4; -; 1.6 (2.5); 1.7; -; 1.5
***ybjS***	b0868	predicted oxidoreductase with NAD(P)_binding domain	2.3
***hns***	b1237	global DNA-binding transcriptional dual regulator H-NS	2.3
***ydeK***	b1510	predicted lipoprotein	2.2
***pdhR aceE aceF lpd***	b0114-6	Pyruvate dehydrogenase decarboxylase component E1, thiamin binding; pyruvate dehydrogenase dihydrolipoyltransacetylase component E2; lipoamide dehydrogenase E3	*1.9*; 2.2; 1.9; 1.5
***yceJ yceI***	b1057-6	YceJ: predicted cytochrome b561; YceI: Periplasmic protein, induced at high pH and by osmotic shock.	1.4; 2.1
***ulaR***	b4191	DNA binding transriptional dual regulator/represses transport and utilization of L-ascorbate	2.0
***vsr***	b1960	DNA mismatch endonuclease of very short patch repair	2.0
*rfaQGPSBIJ * ***rfaY*** * rfaZ-waaU*	b3632-23	Lipopolysaccharide core biosynthesis protein	-; -; -; -; -; -; -; 2.0; -; -
***csrB***	N/A	Regulatory RNA/Carbon storage regulation	1.9
***lgt thyA***	b2828-7	Lgt: prolipoprotein diacylglyceryl transferase, inner membrane; ThyA: thymidylate synthetase	1.9; 1.5
***rffA***	b3791	dTDP-4-oxo-6-deoxy-D-glucose transaminase/Lipopolysaccharide biosynthesis	1.9
***yidZ***	b3711	HTH-type transcriptional regulator	1.9
***spy^Y^***	b1743	Periplasmic protein related to spheroblast formation	1. 75 (39.3)
***cpxP^Y, W^***	b3913	Regulator of the Cpx response and possible chaperone involved in resistance to extracytoplasmic stress (CpxP)	1.5 (3.3)
***pykA***	b1854	Pyruvate kinase II/Anaerobic respiration, glycolysis	−1.9
***agp***	b1002	Periplamic glucose-1-phosphatase.	−1.9
***uspA***	b3495	Universal stress global response regulator	−1.9
***ydeN***	b1498	Uncharacterized sulfatase	−2 (−*2.4*)
***hdhA***	b1619	7-alpha-hydroxysteroid dehydrogenase/lipid, steroid metabolism, belongs to the σ^S^ regulon	−2.0
***adhE^W^***	b1241	Iron dependent aldehyde-alcohol dehydrogenase, pyruvate-formate lyase-deactivase	−2.0
***ygaW***	b2670	Predicted inner membrane protein	−2.0
*gcvP * ***gcvH gcvT^W^***	b2903-5	GcvH: lipoylprotein aminomethyltransferase ; GcvT: tetrahydrofolate dependent subunit/Glycine cleavage complex	-; −2.2; −1.8
***pepT^O^***	b1127	Peptidase T	−2.3
***lamB***	b4036	Outer membrane porin; Phage lambda receptor protein; maltose high-affinity receptor	−*2.5* (−9.8)
***cspD***	b0880	DNA replication inhibitor, induced by stress and glucose starvation, similarity to cold shock protein	−2.5 (−*2.5*)
***gatDCBAZY***	b2091-6	Galactitol specific enzyme IIC and IIB component of PTS	−1.6; −2.6; −2.5; −1.6; −1.9; −1.4
***tnaL tnaA tnaB^O^***	b3707-9	Tryptophan transport and utilization	−*2.1*; −2.9 (−*58.5*); −2.8
***glpA glpB glpC^O^***	b2241-43	Glycerol-3-phosphate dehydrogenase subunits/glycerol degradation	*−2.3*; −1.9; −3.0 (−*8.8*)

a Genes are grouped by putative or known operons (ordered in the direction of transcription). Genes whose expression was found to be significantly modulated are in bold (see Materials and Methods). Genes whose expression was determined by Q-PCR are underlined, and the fold change thus determined is indicated in parentheses after the microarrays value. Genes reported to be CpxR-regulated are indicated by uppercase letters that refer to the concerned study with the following codification: O, reported in [70]; Y, reported in [76]; W, reported in [52].

b b numbers correspond to genes of the first column.

c The fold changes indicate the ratios of gene signal intensities of the Δ*cpxR* strain to the reference signals (see Materials and Methods). Values correspond and follow the order of genes from the first column. The ratio value of genes not significantly modulated is indicated as “-”. Numbers in italics indicate a p-value>0.01 in case of microarray data or >0.05 in case of qRT-PCR.

**Table 7:** Genes whose expression is significantly modulated by the absence of CpxR.

Genes/operon^a^	bnum^b^	Product(s) description/function	Fold change(s)^c^
***yccA^O,Y^***	b0970	Inner membrane protein modulator of HflB (FtsH) protease	−4.2
*fucPIK * ***fucU*** * fucR*	b2801-5	L-fucose mutarotase, fucose catabolisme	-; −4.3; -
***bssR (yliH)***	b0836	Regulator of biofilm formation	−3.4
***flu***	b2000	Antigen 43, outer membrane protein, potential adhesion	−3.2
***ybhT***	b0762	Predicted protein YbhT	−2.9
***yhjV***	b3539	Inner membrane transport protein YhjV	−2.7
***ybaQ***	b0483	Predicted transcriptional regulator	−2.6
***ybeL***	b0643	Conserved protein	−2.6
***smpA^O W^***	b2617	Outer membrane lipoprotein, component of Outer Membrane Protein Assembly Complex	−2.5
***puuC (aldH)*** * puuB (ordL), puuE (goaG)*	b1300-2	γ-glutamyl-γ-aminobutyraldehyde dehydrogenase (putrescine utilization pathway)	−2.5; -, -
***ycgZ ymgA ariR***	b1164-66	Uncharacterized proteins; AriR: regulator of acid resistance influenced by indole	−2.2; −1.9; −*2*
***qseB*** * qseC*	b3025-6	Two component regulatory system, activator of the flagellar regulon	−2.2; -
***ftnB (yecI)^Y^***	b1902	Ferritin-like protein 2	−2.2
***pheM***	b1715	Phenylalanyl-tRNA synthetase (PheST) operon leader peptide (PheM)	−2.0
***yzgL***	b3427	Uncharacterized protein	−2.0
***fliA*** * fliZ * ***fliY***	b1922-20	SigmaF, regulation of flagellar regulon; regulator of FliA; Cystine-binding periplasmic protein	−1.7; -; −1.9
*dppA dppB * ***dppC dppD*** * dppF*	b3544-40	Components of dipeptide ABC transporter	-; -; −1.5; −1.9; -
***csiR (gabC)***	b2664	Regulator of Gab gene expression	−1.9
***ydjF***	b1770	Transcriptional regulator YdjF	1.9
*fryBC * ***ypdF*** * ypdE * ***fryA***	b2387-3	YpdF, aminopeptidase; FryA, fused predicted PTS system enzymes: Hpr component, enzyme I component, enzyme IIA component	-; -; 1.9; -; 1.5
***ycbZ***	b0955	Putative ATP-dependent protease	1.9
***rpsT***	b0023	30S ribosomal protein S20	2.0
*yciG * ***yciF*** * yciE*	b1259-7	YciF: putative structural protein, osmotically induced, belong to the σS regulon	-; 2.2; -
***endA***	b2945	DNA-specific endonuclease I	2.4
***ybcU (borD)***	b0557	Lipoprotein Bor homolog	2.7
***pspF***	b1303	Phage shock protein PspF	3.0
***ligB (yicF)***	b3647	DNA ligase B	3.6

a Genes are grouped by putative or known operons (ordered in the direction of transcription). Genes whose expression was found to be significantly modulated are in bold (see Materials and Methods). Genes reported to be CpxR-regulated are indicated by uppercase letters that refer to the concerned study with the following codification: O, reported in [70]; Y, reported in [76]; W, reported in [52].

b b numbers correspond to genes of the first column.

c The fold changes indicate the ratios of gene signal intensities of strain Δ*cpxR* to the reference gene intensities (see Materials and Methods). Values correspond and follow the order of genes from the first column. The ratio value of genes not significantly modulated is indicated as “-”. Number in italics indicates a p value>0.01.

Altogether our results confirmed just 8 genes/transcription units as regulated by CpxR, out of 33 gene clusters that were assigned by previous *in vivo*, *in silico* and/or *in vitro* studies as members of the CpxR regulon (Tables 6 & 7, [52],[70],[77],[79]). *ppiD*, which encodes a periplasmic peptidyl-prolyl isomerase, was previously proposed to be part of the CpxR regulon. In agreement with Price and Raivio [78], this gene was not affected by *cpxR* overproduction or by deletion, as also confirmed by qRT-PCR (data not shown). In addition, we identified 71 new genes belonging to 49 transcription units that lacked a CpxR box upstream of their promoters [52],[79], which yet were modulated by overproduction or absence of CpxR (Table 6 & 7). Regulation of many of these additional genes can be an indirect consequence of CpxR overproduction or inactivation, and would be expected, as several transcriptional regulators are putative members of the CpxR regulon (Table 6 & 7).

The weak effect of overproducing CpxR on the MG1655 transcriptome could be due to a low basal level CpxR phosphorylation. Alternatively, this could be explained by a dependence upon other regulators, which might be limiting in the absence of an inducing signal. Indeed, most genes thus far identified as being regulated by CpxR are also dependent on other factors, such as σ^E^ or BaeR (Table 4, [58], see also the compilation of CpxR regulated genes in Ecocyc http://ecocyc.org/). This phenomenon would be especially important if the other regulator is a σ factor. Out of 33 gene clusters listed as regulated by CpxR in EcoCyc, 7 depend upon σ^E^, one (the *mdt* operon) depends upon σ^S^ and one upon σ^F^. Many other genes transcribed by σ^70^ are subject to multiple regulations (e.g BaeR, catabolic repression, and other specific regulators) in addition to being regulated by CpxR, pointing to the interdependency of such factors in mounting a full response. In favor of this explanation, *spy* expression, which is regulated independently by CpxR and BaeR [58], responded well to CpxR overproduction (Table 6). In the case of the *acrD* gene and the *mdt* operon, their regulation by CpxR was previously shown to be strictly dependent upon BaeR [58]; accordingly, we observed no effect of CpxR overexpression.

### Comparison of extracytoplasmic stress response pathways

**Figure 1:**
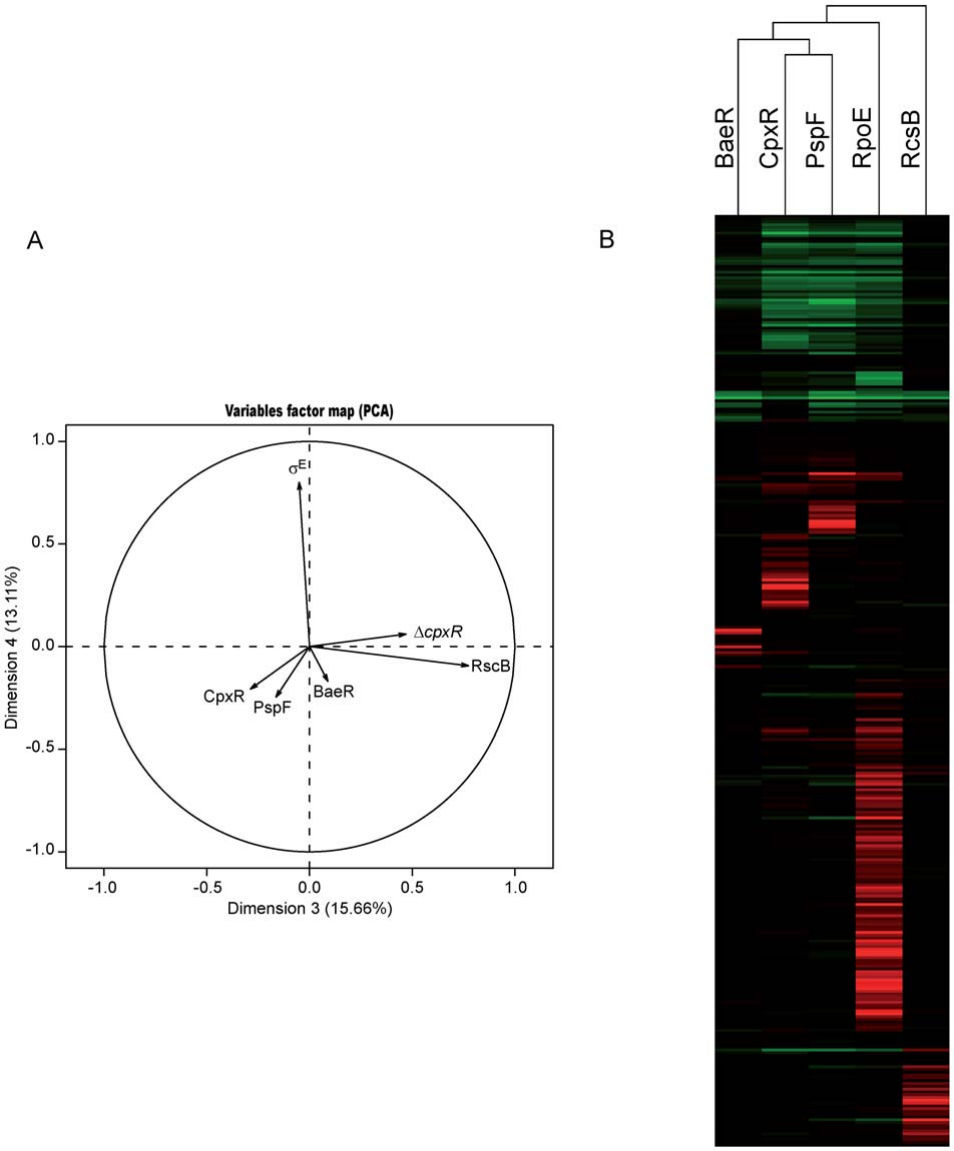
Transcriptome parallel analysis. (A) Correlation circle of the principal component analysis. Arrows indicate the different conditions. Principal component axes 3 and 4 are presented. (B) Hierarchical clustering of genes found to be significantly regulated in all the conditions studied by micro-arrays. Bars in red and green indicate genes that are up- and down-regulated, respectively. Color intensities are proportional to the variation of expression.

Principal components analysis (PCA) is a useful statistical technique that removes noise from complex data sets by reducing the dimensionality and helps discriminate the key factors of variations [80],[81]. PCA has proved useful for finding significant patterns in microarray analyses (see for complete explanation, see [82],[83]). Briefly, given *m* observations (the gene expression ratio) on *n* variables (our 6 different conditions), the goal of PCA is to find *r* significant variables, where *r* is less than *n*, to select the factors that best explain the observed variance in the observations. PCA was used to analyze our microarray data on the mean log-ratio measures obtained, each condition corresponding to a variable (see Materials and Methods). The first dimension accounting for 33% of the variance, could not discriminate between the conditions, whereas the second dimension axis separated CpxR, PspF and *ΔcpxR* conditions from σ^E^, BaeR and RcsB conditions (data not shown). The third and fourth components (accounting together for 29% of the variance) discriminated σ^E^ overproduction and *ΔcpxR* conditions from BaeR/RcsB/CpxR/PspF overproduction conditions (Figure 1A). In the case of σ^E^, both the size of the regulon and the nature of the regulation (*i.e.*, by a σ factor or by transcriptional regulators) could account for this result, whereas in the case of *ΔcpxR*, the result might be explained by the difference in the experimental strategy (deletion vs. overproduction).

**Figure 2:**
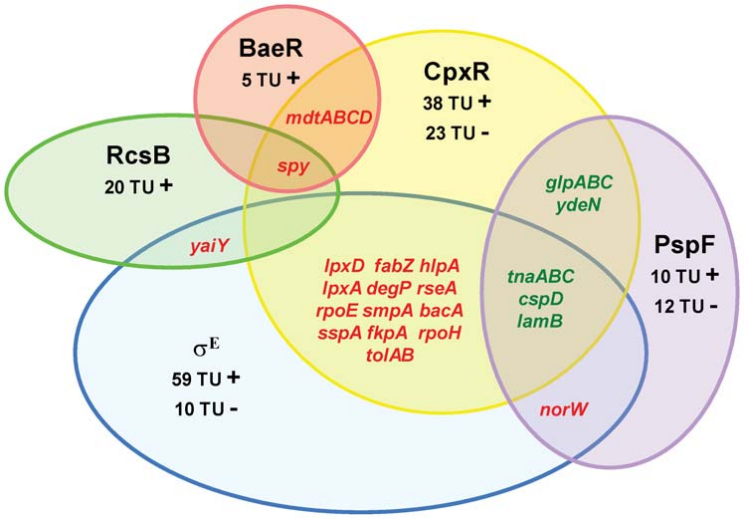
Venn diagram representation of all five extracytoplasmic response pathways. The total number of genes/operons (i.e. transcription units; TU) affected in each condition, positive (+) and negative (−) regulation found in the present study, is indicated for each regulon. The Cpx regulon comprises genes affected by CpxR overproduction or deletion (genes repressed or induced by the absence of *cpxR* are counted + and −, respectively). Only genes that are common to at least two regulons and confirmed by qRT-PCR and/or the literature are listed (red: induced, green: repressed). Genes described in the literature as dependent on CpxR and σ^E^ or BaeR are also included.

A hierarchical clustering that grouped together genes with similar expression patterns was performed on the set of genes differentially expressed in each of the five overexpression conditions (Figure 1B). Results were in agreement with those observed with PCA. The PspF response clustered with the CpxR response, while σ^E^ and RcsB responses were further away. But the main conclusion of this analysis is the striking specialization of each pathway, with very limited overlap between responses (Figure 1B). This is also revealed by a Venn diagram representation showing genes regulated by the extracytoplasmic pathways (Figure 2). Results of this analysis are unexpected, since redundancy is often proposed as an important property of robust networks. In addition, in the case of the extracytoplasmic stress response, redundancy was expected because many genes that are regulated by σ^E^ or BaeR are also regulated by CpxR. Furthermore, several genes regulated by PspF were also affected by CpxR (Figure 2). One surprising finding in our study is that the overproduction of CpxR had a limited effect on many known CpxR regulated genes, in sharp contrast with the situation in the case of RcsB. We propose that in many cases, CpxR acts in conjunction with other regulators, which are limiting in the absence of a stress signal. Hence, rather than controlling in itself specific genes, an important role of CpxR may be to amplify the response promoted by the other regulons. It should be noted that σ^S^ promotes transcription of the *cpxRA* operon [25] and CpxR can cross-talk with the EnvZ-OmpR response [84],[85]. Thus, CpxR may integrate diverse stimuli associated with growth and central metabolism [60]. In view of these analyses, and of some previous results (e.g., [58]), our results suggest that CpxR functions more as a modulator of the other extra cytoplasmic stress responses, especially σ^54E^, BaeR and PspF than as a stand-alone regulator.

σ^E^ appears to be the major regulator, with at least 69 transcription units affected. It is mostly in charge of envelope biogenesis maintenance, especially genes required for synthesis, assembly, and homeostasis of outer membrane proteins and lipopolysaccharides ([57], Table 2). The other responses are more limited and specialized in certain categories of envelope components. PspF is dedicated to maintenance of energy, and has an important role associated with the cytoplasmic membrane in prokaryotes. RcsB affects additional envelope structures such as capsular exopolysaccharide production and O-antigen ([30],[71], Table 3), and BaeR controls the production of several drug export systems that might be important to extrude toxic compounds during an extracytoplasmic stress ([46], Table 4). Hence, each system has its raison d'être in term of restoring various aspects of envelope physiology.

Given the high specialization of each pathway, genes that are regulated by several of these pathways are likely to play a crucial role in cell physiology in response to an extracytoplasmic stress. Although responses are mainly distinct, handful of genes were found to be in common between some of the pathways. For example, the trans-envelope protein components of the Tol-Pal system have a major role in maintaining envelope integrity, which, driven by proton motive force, bring the inner and outer membranes in close proximity [74],[86]. Genes encoding the Tol-Pal system were positively regulated by the σ^E^, Cpx and possibly Psp pathways. In contrast, the *tnaLAB* operon was repressed in by these same pathways (Figure 2). This could be in relation with the fact that *tnaA* encodes tryptophanase, an enzyme that degrades tryptophan and generates indole, itself toxic to the cell and an extracytoplasmic stress inducer. *lamB*, encoding an outer membrane protein was also found to be repressed in several conditions (Figure 2), which may reflect the extra demands imposed on the cell for folding factors controlled by the extracytoplasmic stress regulons [63].

### Concluding remarks

Extensive studies of stress response in *E. coli* have established the existence of several extracytoplasmic pathways, and suggest that expression of numerous genes are affected by more than one of these pathways. These findings suggest redundancy and raise questions concerning the reason for such a multiplicity of pathways. For the first time, we explored all five extracytoplasmic stress response pathways under comparable conditions in *E. coli*. We found that they can be activated simultaneously in response to exogenous or endogenous stimulation. Thus, although activation of a single pathway has been demonstrated experimentally using specific substrates or conditions, our results suggest that natural environmental stimuli provoke bacterial modifications that lead to multiple pathway responses.

To determine the contributions of each stress response pathway, we avoided the use of non-specific inducers, and opted for overexpression of each regulator. Transcriptome analyses show that induction of specific target genes *via* multiple pathways is an uncommon occurrence. Some genes can also be subject to cooperative regulation between different pathways, or to a cascade of pathway responses. In addition, the pathways might cross-talk through transcriptional regulation, as is the case between σ^E^ and σ^S^, and possibly evoked by our results.

Each of for stress response systems (σ^E^, Rcs, Psp and Bae) appears to be specialized in assuring a specific aspect of envelope biogenesis and maintenance, whereas CpxR might have a role as modulator of the response by integrating other endogenous signals. We conclude that all five pathways are needed to mount a full response to extracytoplasmic stress.

## Materials and Methods

### Bacterial strains, plasmids, oligonucleotides, and culture conditions

The *E. coli* strains and oligonucleotides used in this study are listed in supplementary tables S1 and S2. Several plasmids constructed for this study were derived from pZE21 that has a ColE1 replication origin, confers kanamycin resistance and has a P_LtetO-1_ promoter upstream of a multicloning site [87]. pZE21-baeR, expressing *baeR* under the control of P_LtetO-1_, was constructed as follows: The MG1655 *baeR* gene was PCR-amplified using oligonucleotides 450 and 451 (for oligonucleotide sequences, see Table S2). The resulting fragment was digested by KpnI and BamHI and cloned into KpnI-BamHI restricted pZE21-MCS. pZE21-cpxR, pZE21-pspF, pZE21-rcsB and pZE21-rpoE, containing *cpxR*, *pspF*, *rcsB* and *rpoE* were constructed using the same approach as for pZE21-baeR but with 452/453, 454/455, 456/457 and 458/459 oligonucleotide-pairs, respectively. Plasmid pGem-T-easy is a high copy number plasmid conferring ampicillin resistance (Promega, Madison, WI, USA). pGem-T-easy-yedR expressing *yedR* was constructed as follows: MG1655 *yedR* gene was PCR-amplified using oligonucleotides 143 and 144. The resulting fragment was digested by KpnI and BamHI and cloned into KpnI-BamHI restricted pGem-T-easy.

P1 *vir*-mediated transduction was carried out as described [88]. The chromosomal *baeR* gene was deleted by targeted gene substitution using a combination of two published protocols as described [89]. The *baeR* deletion was confirmed by PCR.

Cells were grown in LB broth or on solid LB containing 15 mg.mL^−1^ agar [90]. When necessary, antibiotics were added at the following concentrations: ampicillin 100 µg.mL^−1^, chloramphenicol 30 µg.mL^−1^, kanamycin 20 µg.mL^−1^, and tetracycline 12.5 µg.mL^−1^. Growth of strains containing pZE21-MCS or related plasmids was analyzed as followed: 5 µl of overnight cultures adjusted to OD_600_ of 0.3. Serial dilutions in M9 medium [90] were spotted on solid LB medium containing kanamycin and 0, 2, 10 or 100 ng.mL^−1^ of anhydrotetracycline (aTc), and incubated at 37°C for 24 h.

### Recombinant molecular techniques

Plasmid preparations, DNA cloning and ligation, classical PCR amplification and DNA transformations were carried out according to standard protocols [90] and manufacturers' instructions. Northern blots (see Supplementary Table S2 for information on primers used for probe synthesis) were performed as previously described [90],[91] with 10 to 20 µg of RNA, except that hybridization was performed at 42°C using a NorthernMax prehybridization/hybridization buffer (Ambion, Austin, TX, USA) according to manufacturer's instruction. The *ssrA* gene was used as a reference to normalize RNA quantities in Northern blot experiments.

### β-galactosidase assay

Overnight cultures were diluted in LB broth to an OD_600_ of 0.004. For parallel analyses of inducing and non-inducing growth conditions, cultures were incubated under agitation at 37°C using 96-well culture plates in a total volume of 1 ml. Various growth conditions were investigated: i) standard LB, ii) LB containing ethanol (3 or 5%), iii) 0.5 mM dibucaine, iv) NaCl (0.6 M), v) 5 mM EDTA or vi) indole (2 or 4 mM). The stock indole solution was prepared by dissolving indole in hot LB before use. After five hours, three samples of 200 µl of each culture were taken: One was used to measure OD_600_ in 96-well plates in the Biolumin (Molecular Dynamics) or Chameleon (Bioscan Inc., Washington DC, USA). The two others were used to evaluate β-galactosidase activity according to Miller's protocol adapted to 96-well plate assays [88]. For other β-galactosidase assays, cultures were prepared in individual tubes in 5 mL LB broth under agitation at 37°C. Strains containing pGem-T-easy and related plasmids were grown with 100 µg.mL^−1^ ampicillin. β-galactosidase activities were measured in duplicate from 200 µl samples taken at OD_600_ of 0.4 (exponential phase).

### Gene expression profiling using microarrays

Transcriptome analysis of the effect of transient overexpression of extracytoplasmic stress response regulators was performed with three independent RNA preparations for each of the six biological conditions tested, namely pZE21 (control plasmid), pZE21-baeR, pZE21-cpxR, pZE21-pspF, pZE21-rcsB and pZE21-rpoE. Addition of aTc (for 45 min) to the medium resulted in a 16-, 166-, 225-, 13- and 19- fold increase of baeR, cpxR, pspF, rcsB and rpoE mRNA in strains containing pZE21-baeR, pZE21-cpxR, pZE21-pspF, pZE21-rcsB and pZE21-rpoE respectively, as compared to the reference strain containing the control plasmid, pZE21. Additionally, a Δ*cpxR* strain was included in the study, using four independent RNA preparations. To assess data reproducibility and minimize dye bias effects, one of the samples (two in the case of Δ*cpxR*) was measured with Cy3 instead of Cy5. To ensure robustness and comprehensiveness in data analysis, a reference design was used with an equimolar mixture of all the biological conditions serving as a baseline for the comparisons. Such a design does not require pre-definition of the subgroups for comparison, allows discovery of non-anticipated classes among the samples and is compatible with subsequent additional sampling. Strain MG1655 (Table S1) containing pZE21 and derivative plasmids were grown overnight in LB broth supplemented with kanamycin and diluted in 7 mL LB broth at an OD_600_ of 0.004. After two hours, expression from the P_LtetO-1_ promoter was obtained by addition of 10 ng.mL^−1^ aTc to the medium. After 45 minutes, 7 mL of cold absolute ethanol was added to bacterial cultures (at OD_600_ of about 0.4). Cells were then harvested by centrifugation for 15 min at 3000 *g* and stored at −80°C to prevent RNA degradation. For parental and *cpxR* strain transcriptome analysis, LB overnight cultures were inoculated at OD_600_ of 0.004. When, cultures reached an OD_600_ of 2 (late exponential phase), they were harvested in the presence of cold absolute ethanol and frozen at −80°C. The next steps were carried as for overexpression transcriptome experiments: Cells were lysed and RNA was extracted three times with an equal volume of acidic hot phenol and once with chloroform. RNA was ethanol precipitated, air dried and dissolved in water. RNA integrity was evaluated using RNA 6000 nano chips and the Agilent 2100 Bioanalyzer (Agilent Technologies, Palo Alto, CA, USA) according to manufacturer's instructions. RNA quality control was performed using user-independent classifiers as described [92].

Ten µg of total RNA from each biological condition and the RNA reference mixture were supplemented with RNA corresponding to known sequences to serve as an hybridization control (Spikes, Universal ScoreCard, GE Healthcare), reverse transcribed and labeled using the Superscript Indirect cDNA labeling System (Invitrogen, Carlsbad, CA, USA) according to manufacturer's instruction, except that purification steps were done using the QIAquick PCR mini column system (Qiagen, Hilden, Germany). Labeling efficiency and product integrity was checked according to [93]. For each condition, the hybridization experiment was performed against the reference sample; a mixture of 0.75 µg Cy3- and 0.75 µg Cy5-labeled targets was incubated at 95°C for 3 min in the presence of a 2× Hybridization Buffer (Agilent technologies, Santa Clara, CA, USA). Denatured targets were placed on an *E. coli* v2 whole genome array [63] (ArrayExpress accession: A-MEXP-1516, http://www.ebi.ac.uk/microarray-as/ae/) and hybridized for 17 hours at 60°C, in a rotating oven (6 rpm), using an Agilent hybridization chamber system. The hybridized slides were washed once for 10 min in 2×SCC/0.1% SDS at 50°C and once in 0.5×SCC/0.1% SDS at room temperature, then twice for 5 min in 0.1×SSC at room temperature. Any traces of water were eliminated immediately by air drying with ozone-safe dry air (“canned air”). Slides were scanned using a GenePix 4000B scanner (Molecular Devices, Sunnyvale, CA, USA) at 10-µm resolution. All slides were scanned using 100% laser power; PMT voltages were automatically adjusted using the Genepix Pro 6.0 software acquisition system to obtain maximal signal intensities with <0.005% probe saturation.

The resulting 16 bit images were processed using the GenePix Pro 6.0 image analysis software (v6.0.1.26). Data were processed using the MAnGO software [94], an R script that allows integrated analysis of two-color microarrays. Raw data were normalized using the print-tip loess method [95]. The average Log2 expression ratios were then calculated [log2(pZE21-geneX/REF)−log2(pZE21/REF) = log2(pZE21-geneX/pZE21), where geneX is the gene of interest in the condition of interest, REF the value obtained for X in the case of the pool of all conditions (reference sample), and pZE21 the value obtained in the condition corresponding to the vector alone (biological reference)] and used for all subsequent statistical analyses. MIAME-compliant data [96] were deposited in the ArrayExpress database (http://www.ebi.ac.uk/microarray-as/ae/) under the accession number E-MEXP-2139.

### Data analysis of microarrays

Gene functions were assigned using data from EcoCyc (http://ecocyc.org/) and Uniprot (http://www.uniprot.org/). Adjacent genes coordinately regulated, possibly involved in the same function and separated with a short distance with no apparent terminator were considered as belonging to a putative operon.

#### Dimensionality reduction

To reduce the dimensionality of the expression data set (where the 6 experimental conditions are the variables, and the gene expression measurements are the observations), a principal component analysis (PCA) [80] in gene space, using normalized log-ratio measures, was performed with the R package ADE4 and FactoMineR [97] for calculating three-dimensional projections of the biological samples. We kept the first four components that altogether accounted for over 78% of the explained variance

#### Differential analysis

Statistical comparisons were performed using multiple testing procedures to evaluate statistical significance for differentially expressed genes. A modified *t*-test was computed to measure the significance associated with each differential expression value. An error rate (*p*-value), measuring the risk of false predictions of differentially expressed genes was associated with each test value. A gene expression value was decided to be significantly different under an extracytoplasmic stress response condition when the *p*-value was less than 0.01 (except otherwise mentioned), the signal intensities (A = 0.5log_2_(condition 1×condition 2)) was >6.0, and the expression ratio was ≥1.9 (or ≥1.4 for genes that were part of a putative transcriptional unit containing at least one gene with a fold change ≥1.9), except in the case of the Bae pathway where the fold change cutoff was fixed at 1.4. Genes from prophages were systematically removed from the analysis, as fluctuating intensities may be linked to the presence of numerous paralogs present in the genome and were thus difficult to interpret.

#### Hierarchical clustering

Unsupervised average-linkage hierarchical clustering with uncentered Pearson correlation as a similarity metric was done using Cluster on the gene set defined above [98]. This method leads to an expression matrix such that genes and samples with similar expression patterns are adjacent to each other. Results were visualized with the help of heat-maps and dendrograms using the TreeView program [98].

### Quantitative real-time PCR (qRT-PCR)

One microgram of total RNA was reverse-transcribed in a 30 µl final reaction volume using the High Capacity cDNA Reverse Transcription Kit with RNase inhibitor (Applied Biosystems, Foster City, CA, USA) following the manufacturer's instructions. For each sample, negative reverse transcription reaction was done to verify the absence of genomic contamination in subsequent q-PCR. Primer sequences (see supplementary Table S2) were designed using Primer Express 3.0 software (Applied Biosystems). BLAST searches were performed to confirm gene specificity and the absence of multi-locus matching at the primer site. SYBRGreen q-PCR reactions were performed using the ABI Prism 7900 HT sequence detection system (Applied Biosystems) in 384 well optical reaction plates. 3 µl of cDNA (5 ng/reaction), standard or water (no-template control) were used as template for q-PCR reactions with Fast SYBR Green PCR Master Mix (Applied Biosystems) and primers at 500 nM final concentration. Real-time q-PCR amplifications were carried out (95°C for 20 sec, followed by 40 cycles of 95°C for 1 sec and 60°C for 20 sec, and a final dissociation curve analysis step from 65°C to 95°C). Technical replicate experiments were performed for each biological triplicate sample. The amplification efficiencies of each probe were generated using the slopes of the standard curves obtained by a ten-fold dilution series. The efficiency of the q-PCR amplifications for all of the genes tested was higher than 90%. Amplification specificity for each q-PCR reaction was confirmed by the dissociation curve analysis. Determined Ct values were then exploited for further analysis.

The gene expression levels were analyzed using the relative quantification (delta-Ct method). 16 housekeeping genes were tested and GeNorm and Normfinder functions in Genex 4.3.8 (MultiD, Göteborg, Sweden) were used to select the most stable genes. The geometric mean of 5 housekeeping genes (*dnaQ*, *glnD*, *pcnB*, *uvrB* and *gyrA*) was used to normalize our samples. Data were analyzed with StatMiner 3.0.0 Software (Integromix, Madrid, Spain). Analyses were done with biological replicates and a relative quantification (RQ) value was calculated for each gene with the control group as a reference. RQ values were adjusted according to specific amplification efficiency. A p-value was computed using a moderated t-test to measure the significance associated with each RQ value. Variations were considered statistically significant when the p-value was <0.05 unless otherwise specified in the tables.

## Supporting Information

Table S1 *E. coli* strains used in this study.
(0.11 MB DOC)

Table S2 Primers used for this study.
(0.09 MB DOC)
