# Supplementary material for: Global Analysis of Extracytoplasmic Stress Signaling in Escherichia coli
Source: PLoS Genet. 2009 Sep 18;5(9):e1000651. doi: 10.1371/journal.pgen.1000651 (PMC2731931; doi:10.1371/journal.pgen.1000651)
Supplement: Table S1 — E. coli strains used in this study. (0.11 MB DOC) [file pgen.1000651.s001.doc]

# Supplementary material

## Table S1: *E. coli* strains used in this study

| **Strain** | **Genotype and comments** | **Construction or reference** |
| --- | --- | --- |
| DH5αZ1 | (F^-^) *supE ΔlacU169 ΔargF hsdR17 recA1 endA1 gyrA96 thi-1 relA1* | [1] |
| DY330 | *ΔlacU169 gal490 λcI857 Δ(cro-bioA*) | [2] |
| PhB3309 | DY330 *ΔbaeR*::Km | This study |
| JW5558 | *lacI^q^ rrnBT14 ΔlacZWJ16 hsdR514 ΔaraBADAH33 ΔrhaBADLD78 cpxP*::Km | [3] |
| PND325 | MC4100 λRS88 (*degP-lacZ*) *cpxR::*Spc | [4] |
| SG12020 | C600 *rcsA3* (*rcsA**) *zed-650*::Tn10 | [5] |
| SK1898 | MG1655 *rcsB*::Cm | [6] |
| SG20811 | MC4100 *lon^+^ cpsB::lac rcsC*::Tn10 | [7] |
| DH339 | DH300 *rprA-lacZ ΔrcsD*542::Km | [8] |
| DH303 | MG1655 *ΔlacU169 [*λ* 80 rprA142p::lacZ] rcsF::cat sacB proAB::*Tn10 | [9] |
| RH90 | MC4100 *rpoS*-359::Tn10 | [10] |
| KSJ19 | *clp1*::cat *rprA1*::kan imm21 *rpoS750*::*lacZ* *ΔdsrA .zed3069*:: Tn10, *Δara714* | [11] |
| JW1946 | *lacI^q^ rrnBT14 ΔlacZWJ16 hsdR514 ΔaraBADAH33, ΔrhaBADLD78 yedR*::Km | [3] |
| MC4100 | (F^-^) *lacΔU169 rpsL150 thi-flbB5301 deoC7 ptsF25 relA1* | Laboratory collection |
| lacZ transcriptional fusion containing strains | | |
| TR530 | MC4100 λRS88 [*spy::lacZ*] | [12] |
| PhB3358 | As TR530 *ΔbaeR*::Km | TR530 + P1/PhB3309 |
| TR50 | MC4100 λRS88 [*cpxP::lacZ*] | [13] |
| MC3 | MC4100 λRS88 [*pspA::lacZ*] | [14] |
| GEB658 | MC4100 λRS88 [*rprA142p::lacZ*] | [9] |
| PhB3371 | As GEB658 *rcsB*::Cm | GEB658 + P1/SK1899 |
| PhB3370 | As GEB658 *rcsC*::Tn10 | GEB658 + P1/SG20811 |
| PhB3830 | As GEB658 *rcsD542*::Km | GEB658 + P1/DH339 |
| GEB672 | As GEB658 *rcsF*::Cm | [9] |
| PhB3372 | As GEB658 *djlA*::Km | GEB658 + P1/NM1101 |
| PhB3665 | As GEB658 *rprA*::Km | GEB658 + P1/KSJ19 |
| PhB3649 | As GEB658 *rpoS*::Tn10 | GEB658 + P1/RH90 |
| PhB3854 | As GEB658 *rcsB*::Cm *rpoS:*:Tn10 | PhB3371 + P1/RH90 |
| PhB3586 | As GEB658 *yedR*::Km | GEB658 + P1/JW1946 |
| GEB754 | MG1655 λRS88 [*rprA142p::lacZ*] | MG1655 + P1/GEB658 |
| PhB3822 | As GEB754 *rpoS*::Tn10 | GEB754 + P1/RH90 |
| PhB3853 | As GEB754 *rcsB*::Cm *rpoS*::Tn10 | PhB3822 + P1/SK1899 |
| PhB3861 | As GEB495 *rcsD:*:Km | GEB495 + P1/DH339 |
| PhB3662 | As GEB495 *rcsA* zed-650*::Tn10 | GEB495 + P1/SG12020 |
| PhB3859 | As GEB495 *rcsA* zed-650*::Tn10 *rcsD*::Km | PhB3662 + P1/DH339 |
| CAG16037 | MC1061 Φλ[*rpoH*P3::*lacZ*] | [15] |
| *Genetic background of strains used for transcriptome analysis* | | |
| MG1655 | F^-^ λ^-^ *ilvG*^-^ *rfb-50* *rph-1* | Laboratory collection |
| PhB3319 | MG1655 *cpxR::*Spc | MG1655 + P1/PND325 |
| MG1655Z1 | As MG1655 [(*lacI^q^* *tetR* Spec^R^)] | [16] |
| PhB3924 | MG1655Z1 *cpxP::*Km | MG1655Z1 + P1/JW5558 |

## Strain references

1. Lutz R, Bujard H (1997) Independent and tight regulation of transcriptional units in *Escherichia coli* via the LacR/O, the TetR/O and AraC/I1-I2 regulatory elements. Nucleic Acids Res 25: 1203-1210.

2. Yu D, Ellis HM, Lee EC, Jenkins NA, Copeland NG, et al. (2000) An efficient recombination system for chromosome engineering in *Escherichia coli*. Proc Natl Acad Sci U S A 97: 5978-5983.

3. Baba T, Ara T, Hasegawa M, Takai Y, Okumura Y, et al. (2006) Construction of *Escherichia coli* K-12 in-frame, single-gene knockout mutants: the Keio collection. Mol Syst Biol 2: 2006 0008.

4. Danese PN, Snyder WB, Cosma CL, Davis LJ, Silhavy TJ (1995) The Cpx two-component signal transduction pathway of *Escherichia coli* regulates transcription of the gene specifying the stress-inducible periplasmic protease, DegP. Genes Dev 9: 387-398.

5. Gottesman S, Trisler P, Torres-Cabassa A (1985) Regulation of capsular polysaccharide synthesis in *Escherichia coli* K-12: characterization of three regulatory genes. J Bacteriol 162: 1111-1119.

6. Francez-Charlot A, Castanie-Cornet MP, Gutierrez C, Cam K (2005) Osmotic regulation of the *Escherichia coli bdm* (biofilm-dependent modulation) gene by the RcsCDB His-Asp phosphorelay. J Bacteriol 187: 3873-3877.

7. Painbeni E, Mouray E, Gottesman S, Rouviere-Yaniv J (1993) An imbalance of HU synthesis induces mucoidy in *Escherichia coli*. J Mol Biol 234: 1021-1037.

8. Majdalani N, Gottesman S (2005) The Rcs phosphorelay: a complex signal transduction system. Annu Rev Microbiol 59: 379-405.

9. Castanie-Cornet MP, Cam K, Jacq A (2006) RcsF is an outer membrane lipoprotein involved in the RcsCDB phosphorelay signaling pathway in *Escherichia coli*. J Bacteriol 188: 4264-4270.

10. Lange R, Hengge-Aronis R (1991) Identification of a central regulator of stationary-phase gene expression in *Escherichia coli*. Mol Microbiol 5: 49-59.

11. Majdalani N, Chen S, Murrow J, St John K, Gottesman S (2001) Regulation of RpoS by a novel small RNA: the characterization of RprA. Mol Microbiol 39: 1382-1394.

12. Raivio TL, Laird MW, Joly JC, Silhavy TJ (2000) Tethering of CpxP to the inner membrane prevents spheroplast induction of the cpx envelope stress response. Mol Microbiol 37: 1186-1197.

13. Raivio TL, Silhavy TJ (1997) Transduction of envelope stress in *Escherichia coli* by the Cpx two-component system. J Bacteriol 179: 7724-7733.

14. Bergler H, Abraham D, Aschauer H, Turnowsky F (1994) Inhibition of lipid biosynthesis induces the expression of the *pspA* gene. Microbiology 140 ( Pt 8): 1937-1944.

15. Mecsas J, Rouviere PE, Erickson JW, Donohue TJ, Gross CA (1993) The activity of sigma E, an *Escherichia coli* heat-inducible sigma-factor, is modulated by expression of outer membrane proteins. Genes Dev 7: 2618-2628.

16. Bohn C, Collier J, Bouloc P (2004) Dispensable PDZ domain of *Escherichia coli* YaeL essential protease. Mol Microbiol 52: 427-435.
