# Supplementary material for: Global Analysis of Extracytoplasmic Stress Signaling in Escherichia coli
Source: PLoS Genet. 2009 Sep 18;5(9):e1000651. doi: 10.1371/journal.pgen.1000651 (PMC2731931; doi:10.1371/journal.pgen.1000651)
Supplement: Table S2 — Primers used for this study. (0.09 MB DOC) [file pgen.1000651.s002.doc]

## Table S2: Primers used for this study.

| *Primers used for baeR gene disruption* | | |
| --- | --- | --- |
| **Name** | **Target** | **Sequence** |
| 256 | *baeR* | gtaagcattacagtagagttaccgctggaacgggatttacagagagaagtatgagtgtaggctggagctgcttc |
| 257 | *baeR* | cggatctggtctgaacgccatctccggctaacaaaataatgtcgctaaaaatgaatatcctccttagtt |
| *Primers used for plasmid constructions* | | |
| **Name** | **Target** | **Sequence** |
| 450 | *baeR* | GGGGTACCATGaccgagttaccaatcgacg |
| 451 | *baeR* | GCGGGATCCaataatgtcgctaaaacta |
| 452 | *cpxR* | GGGGTACCATGaataaaatcctgttagttg |
| 453 | *cpxR* | GCGGGATCC***cggtttagctgcctatca*** |
| 454 | *pspF* | GGGGTACCATGgcagaatacaaagata |
| 455 | *pspF* | GCGGGATCCtgtattgctcaacttcgcta |
| 456 | *rcsB* | GGGGTACCATGaacaatatgaacgtaa |
| 457 | *rcsB* | GCGGGATCC***atctggcctacaggtgatta*** |
| 458 | *rpoE* | GGGGTACCATGagcgagcagttaacgg |
| 459 | *rpoE* | GCGGGATCCcagtatcccgctatcgtca |
| 143 | *yedR* | GGTACCGTGGAAAAATGTGACTTTTA |
| 144 | *yedR* | GGATCCTTATTCATAGCGTCTGCTAC |
| *Primers used for Northern-blot probes* | | |
| **Name** | **Target** | **Sequence** |
| 343 | *ssrA* | tcatggcgctcataaatctg |
| 344 | *ssrA* | gcgatctcttttgggtttga |
| 827 | *ynjA* | gccgcttagcaaaattcctt |
| 776 | *ynjA* | tcggcgactttcatactggt |

| *Primers used for qRT-PCR* | | |
| --- | --- | --- |
| **Target** | **Forward** | **Reverse** |
| *argD* | ACCTCTGCGAAAGCGTTAGG | GGATGAAACGCAGAAGCAATTT |
| *asnB* | CCAACAAAAAACCCACGTACTG | AACTGGTAACGATCGCCATATTC |
| *betA* | TTTGACTTCCGCACCCAGAT | TCCGTTTCATAGGCCCAGTT |
| *dnaQ* | ACCGAAACCACCGGTATGAAC | ACCACTTCAACGGCACCAAT |
| *dxr* | TGTCACTCGCATGGTAGAACAGT | GAAGTTTCGCACTCGCTTCAT |
| *entD* | GAGTTCGATCCGGCGAATTT | TTACGTCCAGCGTGTTGCA |
| *glnD* | TGCAAAACACGCCGGATAT | GATGTTATCCATGCGCAGTAGTG |
| *lacI* | GACTGGGCGTGGAGCATCT | CGCCGAGACAGAACTTAATGG |
| *pcnB* | GAACTGCAGCGTCTGGTGAA | AGCTCGTTGAGCATCCCTTTT |
| *phoA* | AAGCCCGCAGTCACCTGTAC | AATGGCTTTGTCGGTCATCTG |
| *proA* | GTGAAATGCTGCGTATGGATAAAT | TTCACGGCACAGTTTATGCAA |
| *recA* | TGCGTTTATCGATGCTGAACA | GAGCACAGCAGGTTGTCGATATC |
| *rnhA* | GCATCAAATCAAATGGGAATGG | CGGCACGAGCCAGTTCAT |
| *sulA* | AGCTCTCCCCTTGCCACACT | CAACCAACCGATCACCACACT |
| *uvrB* | TTTCCACTATTCCACGTTTTACCA | TGATCTCCTCCATCGCCTGTA |
| *gyrA* | GCGTGCGTGATGGTCTGTAC | CGTGCTCAAGACCGGTCAGT |
|  |  |  |
| *amiA* | GTTTGGCTGCCCTGACGTT | GCTGTGTCCGTTGCTGGTTT |
| *ariR* | CGCAGTTCGGGTAATTTGTTAGA | CGCCTGAAAGCATTAAATTGG |
| *bioC* | GGCAGCCGCACACTATGAG | TTACGCTGTGGAAGCATTGC |
| *bssR* | CCATGATTGCCCGACTGTTA | GCTCTGGATACTCAGCGTTAAGC |
| *ccmA* | ACGAGCCTTTTACCGCGATT | CCCCCCCTGCTCCGTAT |
| *cpxP* | AGTTACCGCTGCCGTCATG | AGTTATCGCCTGAACCGACTTC |
| *cspD* | TGGTTCAACAATGCCAAAGG | CGTTCTGTAACCATCCATCTGAAT |
| *cvpA* | TAATCGCGGTGATTGCTTTTT | CCATGTCACCAGCGATAACG |
| *htrA (degP)* | TGAGCGATGGCCGTAAGTTC | CGGGTTCTGGATTTGGATCA |
| *djlA* | GATAAAGCCCGTAGCCGTAAAA | TCAAAAGTGGTGGCAAAAAACA |
| *dsbA* | AAAGTGACTGTTCCGCTGTTTG | CGTTGATAAATACATCGCGGATAT |
| *flu* | CCACCTGGAATATCCCCGATA | TGCGGGTGGAGGTGAAA |
| *glpC* | TCACACTCCGTGCCATATGG | ACTCAAGCCCCGGGATGT |
| *idnR* | CACCATCAGGACGGTGAAATT | CCAGCCGTTCTCCCTGTACA |
| *lamB* | GGCCTTCCGTGAAGCAAAC | AGCGCTTACCTGCCCAGAT |
| *mdtF* | CGGGACGTTTGCGATCTT | ACCCTATCGCCAGCACCAT |
| *mviN (murJ)* | GCTACCGCACCTGAAGAAGATC | TCACCACGCGCATTGCT |
| *napA* | CGATCCGTATCCGACAGTCAGT | CGTAAGCGCCCTCTTTCTCTAC |
| *norW* | GCAAAGCGGTCACGCTAATC | CTGCAAGCGGCTGCTTACTT |
| *ompW* | GCGGCTTTGGCAGTAACAAC | GAACCTGCACGCATAAAAAATTC |
| *ppiD* | TTGCCGACGCTATCTTTAACG | CGCCGTCTACGGTGATGAT |
| *recB* | TTGCGCCTGTTACTTGGACTAG | AAGGTGACCACCAGCAGTTCTT |
| *smpA* | CTGATGTCCGATCCATTTGGT | CGTTTGCTGAGTTACACCTTCATG |
| *spy* | GGCCAGCGTGACCAGATG | TCGAAGGTATCGCTGGCAAT |
| *tnaA* | TGGATCGCAGCAAAATGGT | GCACGGTACAGCCGTTGAT |
| *tolA* | GCTCAAGCGGGCGATAATTA | TCTCATCGAACGAACTCCAGATC |
| *yaiY* | CGCCCGGCGTCTATGAG | GGTGCCGACACCTAAACCAA |
| *ydeN* | GTTAACCGATGAGGCAATTGG | GCATTATAAGCCAGGTAAAGCATAAA |
| *ygdG* | TGCGCTCGATCAGCTCATTA | AGCCGCTACTGCGGTTTTC |
| *yhaI* | TATGCTTTATTTGCTGGCAACCT | CACCTGAACGGTCGGTATCG |
| *yiaF* | GATGCGTAGCGGTGAACGT | TCGCGTAATCAGAGACAAAAGG |
